# Characterization, thermostable mechanism, and molecular docking of a novel glucose-tolerant *β*-glucosidase/*β*-galactosidase from the GH1 family isolated from Rehai hot spring

**DOI:** 10.3389/fmicb.2025.1559242

**Published:** 2025-04-11

**Authors:** Yu-Ying Huang, Dan Zhu, Li-Quan Yang, Maite Ortúzar, Zheng-Feng Yang, Zhi-Hua Lv, Kai-Qing Xie, Hong-Chen Jiang, Wen-Jun Li, Yi-Rui Yin

**Affiliations:** ^1^College of Agriculture and Biological Science, Dali University, Dali, China; ^2^College of Marine Science, China University of Geosciences, Beijing, China; ^3^State Key Laboratory of Biocontrol, Guangdong Provincial Key Laboratory of Plant Resources and Southern Marine Science and Engineering Guangdong Laboratory (Zhuhai), School of Life Sciences, Sun Yat-sen University, Guangzhou, China; ^4^Xizang Key Laboratory of Plateau Fungi, Institute of Plateau Biology of Xizang Autonomous Region, Lhasa, China; ^5^Cangshan Forest Ecosystem Observation and Research Station of Yunnan Province, Dali University, Dali, China; ^6^Co-Innovation Center for Cangshan Mountain and Erhai Lake Integrated Protection and Green Development of Yunnan Province, Dali University, Dali, China; ^7^State Key Laboratory of Biogeology and Environmental Geology, China University of Geosciences, Wuhan, China

**Keywords:** Tengchong Rehai, thermostable, glucotolerant, *β*-glucosidase, *β*-galactosidase

## Abstract

**Introduction:**

As a renewable alternative to fossil fuels, second-generation bioethanol production relies heavily on efficient lignocellulose conversion, with *β*-glucosidase playing a critical role.

**Methods:**

This study focused on the *β*-glucosidase gene *y50bg4* discovered in the Tengchong Rehai metagenome. The recombinant enzyme Y50Bg4 was obtained through PCR amplification, cloning, and expression. It was subsequently separated and purified using a Ni-NTA affinity chromatography column, and its enzymatic properties were analyzed.

**Results:**

Enzymatic characterization revealed that Y50Bg4 efficiently hydrolyzes substrates like cellobiose, pNPGlc, and lactose. Y50Bg4 achieved optimal activity at 60°C and pH 6.0, maintaining 100% stability after 2 h of incubation at 60°C. The residual activity remained above 60% after 24 h of incubation across a pH range of 4.0 to 10.0. Kinetic constants analysis showed *K*_m_ values of 4.69 mg/mL for cellobiose and 0.53 mM for pNPGlc, with *V*_max_ values of 39.71 μmol/min/mg and 20.39 μmol/min/mg, respectively. Furthermore, the enzyme exhibits exceptional glucose tolerance, with Y50Bg4 retaining over 80% of its activity even at a glucose concentration of 3,000 mM. In practical applications, Y50Bg4 can work synergistically to degrade corn straw when combined with commercial cellulase. When Y50Bg4 (0.05 mg/mL) was added to the commercial cellulase reaction system, the glucose yield from corn straw increased by 11.6% after a reaction period of 24 h at 50°C. The results indicate that Y50Bg4 exhibits the activities of both *β*-glucosidase and *β*-galactosidase. Molecular docking and kinetic simulations revealed that Y50Bg4 has a higher affinity for cellobiose than for lactose and identified structural regions (residues 325–350 and 390–410) that contribute to its thermal stability.

**Discussion:**

These findings highlight the potential of Y50Bg4 for industrial applications in bioethanol production and cellulose hydrolysis. In summary, Y50Bg4, with its exceptional enzymatic properties, presents significant application value and market potential in industrial sectors such as bioethanol production and cellulose hydrolysis.

## Introduction

Lignocellulose is the most abundant and cost-effective renewable resource on Earth. Its main component is cellulose, which is a sustainable and environmentally friendly material for producing high-value industrial products, such as biofuels and chemicals. It plays a crucial role in addressing energy and environmental crises (Chimtong et al., 2011; Bilal et al., 2020) The complete hydrolysis of cellulose is driven by a combination of three main cellulases: endoglucanases (EC 3.2.1.4), exoglucanases (EC 3.2.1.91), and *β*-glucosidases (EC 3.2.1.21). Among these, *β*-glucosidase is the most important component of the cellulase system and acts as the key enzyme in cellulose hydrolysis (Bhatia et al., 2002). It degrades cellobiose and fibrooligosaccharide into glucose during cellulose saccharification (Bhatia et al., 2002; Lynd et al., 2002). However, insufficient catalytic activity of *β*-glucosidase can hinder glucose production, leading to the accumulation of cellobiose, which in turn impairs glucose formation. As a depolymerase inhibitor, cellobiose also suppresses the activity of endoglucanase and exoglucanase (Dekker, 1986). Therefore, *β*-glucosidase has become a key rate-limiting enzyme in the overall process and a significant bottleneck for the biodegradation of cellulose to glucose. Many *β*-glucosidases reported to date are very sensitive to glucose, with glucose inhibition constants (*K*_i_) typically below 100 mM. In recent years, glucose-tolerant *β*-glucosidases have become a major research focus due to their potential to enhance the saccharification process of lignocellulosic materials (Fang et al., 2010). In addition, most natural enzymes can only react under mild conditions, which limits their industrial applicability. In contrast, thermostable *β*-glucosidases, known for their resistance to high temperatures, are gaining significant attention in industrial production. These enzymes are considered an ideal catalyst for natural glycoside conversion due to their robust properties, including heat stability (Yenenler and Sezerman, 2016; Zhang et al., 2017). For example, cellulose degradation typically occurs at temperatures above 50°C and can require extended reaction times (sometimes over 100 h). In these processes, the inclusion of thermostable *β*-glucosidases improves catalytic efficiency, reduces substrate degradation, and lowers both energy consumption and environmental impacts (Haki and Rakshit, 2003; Liu et al., 2012). Therefore, finding efficient, thermostable, and glucose-tolerant *β*-glucosidases is essential to advance both research and industrial applications of cellulase technology.

Recent studies have identified several *β*-glucosidases from hot spring environments that exhibit both thermostability and glucose tolerance (Kaushal et al., 2021; Sinha et al., 2021; Zhu et al., 2024). However, most microorganisms from natural environments cannot be easily cultured in pure culture in the laboratory. To overcome this limitation, metagenomic techniques, which enable the exploration of environmental microbial genetic resources, have been successfully used to clone enzymes of significant industrial value (Richardson et al., 2002; Green and Keller, 2006). With the increasing importance of *β*-glucosidases and their various biotechnological applications, many efforts have been made to isolate and characterize novel enzymes from different sources to improve their performance.

In this study, we screened a *β*-glucosidase gene (named *y50bg4*) from the metagenome of the Tengchong Rehai hot spring, based on its functional potential. The gene was induced and expressed in *Escherichia coli* DH5α, and the recombinant enzyme *β*-glucosidase Y50Bg4 was purified for further analysis. Y50Bg4, which belongs to the GH1 family, was characterized for its enzymatic properties, and its thermostable mechanism was investigated through molecular dynamics simulation and molecular docking studies. The results showed that Y50Bg4 exhibits appreciable thermal stability, a broad pH range, and glucose tolerance, positioning it as an ideal enzyme candidate for applications such as hemicellulose degradation and other industrial processes.

## Materials and methods

### Sample collection, enrichment culture, and metagenomic sequencing

Sediment and spring water samples were collected from hot springs in the Rehai area, Tengchong County, Baoshan City, Yunnan Province, Southwest China (24.947063°N, 98.440565°E), and stored at room temperature in the laboratory for enrichment culture. A 5 g sediment sample and 100 mL of spring water were supplemented with 1 g of cellobiose to the MSM (Minimal Salt Medium) as a carbon source and incubated at 50°C for enrichment culture. Samples were collected every 24 h for determination of total sugar content until depletion, with enriched samples obtained by centrifugation at 4,000 × *g*. Metagenomic DNA was extracted using the MOBIO DNeasy PowerSoil Kit (USA) according to the manufacturer’s instructions, and metagenomic sequencing was performed on a HiSeq2500 platform at GENWIZ (Suzhou). The IMG server^1^ was used to analyze the sequences, and gene functions were predicted using KEGG (Nakaya et al., 2013), COG (Tatusov et al., 2001), and Pfam (Finn et al., 2014) databases.

### Gene sequence prediction and analysis of Y50Bg4

Based on functional predictions, a gene encoding *β*-glucosidase (named *y50bg4*) was identified from metagenomic data. The nucleotide sequence was submitted to GenBank under the accession number PQ827568. The nucleotide and amino acid sequences of Y50Bg4 were aligned using the BLASTx and BLASTp programs^2^, respectively. Signal peptide prediction was done using SignalP^3^, and primary structure analysis and molecular weight prediction were determined with the ExPASy tool^4^.

Phylogenetic analysis was conducted using MEGA 7 software (Kumar et al., 2016). To determine the optimal substitution model for sequence alignment, a model selection test was performed, which identified the WANG model as the most appropriate based on its lowest Akaike Information Criterion (AIC) and Bayesian Information Criterion (BIC) scores. Maximum likelihood (ML) phylogenetic reconstruction was then carried out using this selected model, with nodal support assessed through 1,000 bootstrap replicates. To enhance the phylogenetic resolution of Y50Bg4 within the *β*-glucosidase family, we incorporated representative sequences from three glycoside hydrolase families (GH1, GH3, and related glycoside hydrolase families) in the phylogenetic tree construction. All reference sequences were obtained from the NCBI protein database^5^.

### Homology modeling of Y50Bg4

The 3D structure of Y50Bg4 was predicted using the AlphaFold2 server^6^ (Jumper et al., 2021; Varadi et al., 2022) and visualized in PyMOL (Delano, 2002). Model quality was assessed using the pLDDT score, which ranges from 0 to 100. The Y50Bg4 structure was compared with *Thermomicrobium roseum β*-glucosidase B9L147, and multiple sequence alignment was conducted using MEGA 7 and visualized and analyzed with ESPript^7^.

### Cloning, expression, and purification of y50bg4

Specific primers (*y50bg4*)-F: (CATCATCATCATCATCATGAAAATAGATGGAGGGCTAAAATGT) and (*y50bg4*)-R: (GTGCTCGAGTGCGGCCGCAAGTTCTTTATACTTTTTAATTATTTC) containing restriction sites were designed abased on the *y50bg4* DNA sequencing using Primer Premier 5.0 to amplify the full-length *y50bg4* fragment. The underlined sequence corresponds to the homologous recombination fragment for insertion into the pSHY211 vector (Yin et al., 2023), which had been previously digested with *EcoR I* and *Hind III*. The complete open reading frame (ORF) of *y50bg4* was amplified with the high-fidelity enzyme Gold Mix (Beijing Tsingke Biotech Co., Ltd., China) using the following PCR protocol: pre-denaturation at 95°C for 3 min, followed by 30 cycles of denaturation at 98°C for 20 s, annealing at 65°C for 30 s, extension at 72°C for 45 s, and a final extension at 72°C for 5 min. The resulting PCR product was inserted into the pSHY211 vector using the pEASY-Uni Seamless Cloning and Assembly Kit (TransGen Biotech, China), generating the expression plasmid pSHY211-y50bg4. *E. coli* DH5α was used for *y50bg4* gene cloning and expression, with the bacteria cultured in LB medium containing 50 μg/mL kanamycin. DNA isolation and purification were performed using kits from Sangon (China).

For expression of Y50Bg4, the recombinant plasmid was transformed into *E. coli* DH5α. The transformants were cultured in 200 mL of LB liquid medium with 50 μg/mL of kanamycin at 37°C with shaking at 200 rpm for 8 h. The culture was then incubated at 20°C with shaking at 200 rpm for 24 h. Cells were harvested by centrifugation at 10,000 × *g* for 15 min at 4°C, and then disrupted by sonication. The lysate was centrifuged at 12,000 × *g* for 30 min at 4°C, and the supernatant was purified using a Ni-chelating affinity column (Histrap, TransGen Biotech, China) as described by Yin et al. (2017). The purified recombinant *β*-glucosidase Y50Bg4 was analyzed by 12% SDS-PAGE (sodium dodecyl sulfate-polyacrylamide gel electrophoresis), with protein bands visualized by Coomassie Brilliant Blue dye R-250 staining. The protein concentration was determined using the Bradford Protein Assay Kit (order number C503031, Sangon Biotech, China), with bovine serum albumin as the standard (Bradford, 1976).

### Determination of *β*-glucosidase activity

Enzyme activity was measured using the glucose oxidase-peroxidase assay kit with cellobiose as substrate (Yin et al., 2018). A 10 μL aliquot of 10-fold diluted enzyme solution after purification by Ni-NTA affinity chromatography was added to 90 μL of a buffer containing 1% (w/v) cellobiose. To minimize the occurrence of non-specific side reactions, such as substrate degradation or the acceleration of unintended chemical reactions, the enzymatic reaction was terminated immediately after 30 min under optimal conditions by transferring the samples to a pre-cooled −80°C environment and freezing them for 2–3 min. The transfer time was strictly limited to 30 s to reduce the impact of temperature fluctuations on enzyme activity. To quantify enzyme activity, 10 μL of the reaction mixture was added to a 96-well culture plate with 200 μL of glucose oxidase-peroxidase assay kit buffer. The mixture was incubated at 37°C for 10 min, and absorbance at OD490 nm was measured. One unit (U) of *β*-glucosidase activity was defined as the amount of enzyme required to release 2 μmol of glucose from cellobiose per minute. All experimental conditions were strictly controlled to ensure consistency across replicates.

### Effect of temperature and pH on *β*-glucosidase activity

The optimum temperature was determined by measuring enzyme activity across a temperature range of 30–85°C at the optimal pH. The optimal pH was assessed at the identified optimal temperature using sodium citrate buffer (pH 3.0–8.0) and glycine-NaOH buffer (pH 8.0–10.6). Results were expressed as the relative activity compared to the values obtained at the optimal temperature and pH. For thermal stability testing of Y50Bg4, the enzyme was pre-incubated at 50, 55, and 60°C for different time intervals (0-180 min). Residual enzyme activity was subsequently measured under optimal conditions. pH stability was assessed by incubating the enzyme in the specified buffers at 4°C for 12 and 24 h, and the residual activity was measured under standard conditions.

### Effect of glucose on *β*-glucosidase activity

To assess the effect of glucose on the enzymatic activity of Y50Bg4, p-nitrophenyl-*β*-d-glucopyranoside (pNPGlc) was used as the substrate. A 10 μg protein was added to 200 μL of reaction mixture containing 2.5 mM pNPGlc (Sigma, St. Louis, MO, United States). The reaction was incubated for 5 min at the optimal temperature, then terminated by adding 450 μL of 1 M Na_2_CO_3_. The release of *p*-nitrophenol was measured by monitoring the absorbance at 405 nm, using *p*-nitrophenol (Sigma, St. Louis, Missouri, United States) as a standard. To evaluate the effect of *β*-glucose on enzyme activity, different concentrations of *β*-glucose (0–3 M) were added to the reaction mixture. Relative enzyme activity was determined by comparing it with a control reaction that did not include glucose.

### Determination of the effects of metal ions and organic reagents on *β*-glucosidase activity

The impact of metal ions and organic reagents on *β*-glucosidase activity was investigated by adding various metal ions (K^+^, Mg^2+^, Fe^3+^, Ca^2+^, Co^2+^, Ni^2+^, Mn^2+^, Pb^2+^, Cu^2+^, Zn^2+^, and Ag^+^) to the enzyme solution at final concentrations of 1 mM and 10 mM. Additionally, the effects of organic reagents, including Ethylenediaminetetraacetic acid (EDTA), Tween-80, Ethanol, Sodium Dodecyl Sulfate (SDS), and Phenyl Methyl Sulfonyl Fluoride (PMSF), were tested at final concentrations of 0.1 and 1%. The enzyme was incubated at 60°C, and the residual activity was measured after 30 min of treatment.

### Determination of substrate specificity and kinetic parameters of Y50Bg4

To determine the substrate specificity of Y50Bg4, a variety of substrates were tested, including cellobiose, p-nitrophenyl-*β*-d-glucopyranoside (pNPGlc), p-nitrophenyl-*β*-d-galactopyranoside (pNPGla), *α*-lactose, sucrose, maltose, gentiobiose, d-(+)-melibiose hydrate, d-(+)-trehalose dihydrate, raffinose, microcrystalline cellulose (Avicel), beechwood xylan, carboxymethylcellulose sodium (CMC-Na), corncob xylan, and bagasse xylan, all at a concentration of 1% (w/v). Cellulase and xylanase activities were quantified using the 3,5-dinitrosalicylic acid assay. One unit (U) of enzyme activity was defined as the amount of enzyme required to release 1 μmol of reducing sugar per minute.

The kinetic properties of recombinant Y50Bg4, including *K*_m_ and *V*_max_, were evaluated in sodium citrate buffer (pH 6.0) at 60°C, using cellobiose and pNPGlc as substrates. Substrate concentrations ranged from 0 to 20 mg/mL for cellobiose and 0–2 mM for pNPGlc. The kinetic parameters were determined through non-linear regression analysis using the Michaelis–Menten equation in GraphPad Prism 5, yielding the *K*_m_ and *V*_max_ values. These results were further validated by Hanes-Woolf plots.

### Hydrolysis of corn stover by *β*-glucosidase

Ten gram of corn stover were ground and sieved through an 80-mesh sieve. The stover was then boiled in 100 mL of hot water for 30 min, filtered, and dried at 80°C. A 0.2 g aliquot of the pretreated corn was added to 1 mL of buffer (pH 5.6) along with 0.2 mg of cellulase (Sangon Biotechnology, China) and 0.03 mg of Y50Bg4. The mixture was incubated at 50°C, and samples were collected at 0, 1, 2, 3, 4, 5, 6, 7, 8, 18, and 24 h post-reaction. Glucose concentration in the reaction mixture was determined using a glucose oxidase assay kit. A control reaction without enzyme was also included.

### Molecular dynamics simulations

A similarity search was performed using BLAST to identify an ambient *β*-glucosidase ThBGL1A with high homology to Y50Bg4, which was derived from *Thermobifida halotolerans* YIM 90462^T^. Molecular dynamics simulation was performed to analyze the structural stability and conformational flexibility of both Y50Bg4 and ThBGL1A. All simulations were conducted with identical steps and parameters. MD simulations were performed using GROMACS software, based on the protein structure and topology files obtained previously (Abraham et al., 2015). For the construction of the simulated environment, protein molecules were placed in a suitable solvent model to simulate their real state in the physiological environment. At the same time, an appropriate amount of counterions was added to make the simulation system electrically neutral. For the simulation parameter settings, we adopt a series of parameters that have been widely verified and optimized. The time step was set to 2 fs to ensure the simulation accuracy while effectively controlling the consumption of computational resources. The temperature is set at 300 K to simulate the environmental conditions close to the physiological temperature and to ensure that the structure and kinetic behavior of proteins are more realistic. The pressure was maintained at 1 bar to simulate the normal physiological pressure environment. The root means square deviation (RMSD) of the backbone and the root mean square fluctuation (RMSF) of C*α* per residue during the simulations were calculated and compared using the GROMACS tools (gmx rms and gmx rmsf).

### Molecular docking

The 2D structures of the small molecule ligands, α-lactose and cellobiose, were downloaded from PubChem^8^, in SDF format and converted to 3D structures using ChemDraw 3D software, followed by energy minimization. The receptor Y50Bg4 and the ligands α-lactose and cellobiose were docked using AutoDock Vina software. The protein Y50Bg4 and the ligand molecules were pre-processed, and docking parameters were set with the following box dimensions: center_x = −0.133, center_y = −1.194, center_z = 0.019, size_x = 36.0, size_y = 28.5, size_z = 29.25. The docking simulations were executed, and optimized conformations for the different ligands were obtained.

### Statistical analysis

All experiments were conducted in triplicate unless otherwise noted. Results were expressed as mean ± standard error of the mean (SEM). Statistical analyses were performed using SPSS version 20.0. One-way ANOVA followed by Tukey’s *post hoc* test was used for comparison among multiple experimental groups. A *p*-value of less than 0.05 was considered statistically significant, with a *p*-value less than 0.01 (**) indicating highly significant differences and *p*-values less than 0.05 (*) indicating significant differences.

## Results

### Metagenomic sequencing and sequence analysis of Y50Bg4

Metagenomic sequencing of the hot spring enrichment from Tengchong Rehai yielded a comprehensive dataset comprising 6.72 Gbp and including 28,299 contigs longer than 1,000 bp. This dataset enabled the identification of 192,214 unique genes, among which 44 were predicted to be *β*-glucosidase genes. Based on gene integrity and sequence novelty, a novel gene *y50bg4*, encoding a GH1 family *β*-glucosidase, was identified from a Tengchong hot spring metagenomic data. Y50Bg4 is predicted to contain a glycoside hydrolase (GH1) structural domain (Pfam 00232; COG2723 *β*-glucosidase). The gene comprises a 1,338 bp open reading frame encoding a polypeptide of 446 amino acids. Based on EXPASY calculations, the predicted isoelectric point (*pI*) and molecular weight (*Mw*) are 6.4 and 52.7 kDa, respectively.

**Figure 1:**
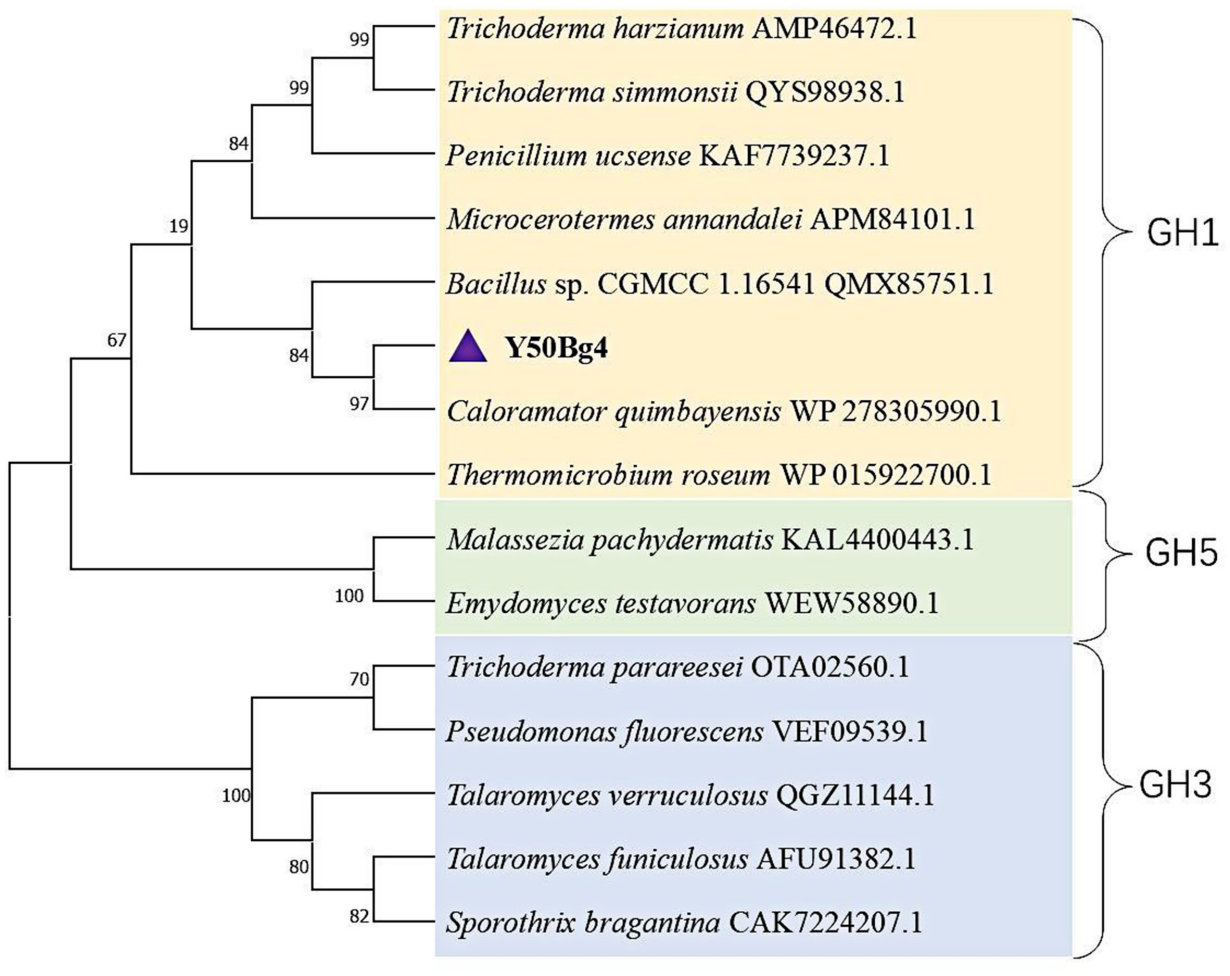
Phylogenetic analysis of Y50Bg4 and related *β*-glucosidases. The maximum likelihood phylogenetic tree was constructed using the WANG model with 1,000 bootstrap replicates. Branches are color-coded to represent different glycoside hydrolase families: yellow for GH1 family *β*-glucosidases, blue for GH3 family *β*-glucosidases, and green for GH5 family glycoside hydrolases. Y50Bg4 (highlighted in purple) clusters with *β*-glucosidase from *Caloramator quimbayensis* (80.65% sequence similarity) and is closely related to other GH1 family members. Bootstrap values (>50%) are shown at branch nodes.

Sequence alignment analysis revealed that Y50Bg4 exhibits 80.65% sequence similarity with *β*-glucosidase from *Caloramator quimbayensis* (NCBI accession: WP278305990.1). Phylogenetic reconstruction demonstrated that these two enzymes form a distinct cluster, with Y50Bg4 showing close evolutionary relationships to other characterized GH1 family *β*-glucosidases (Figure 1). These findings provide strong evidence supporting the classification of Y50Bg4 within the glycoside hydrolase family 1 (GH1). Multiple sequence alignment confirmed conserved regions typical of GH1 family *β*-glucosidases (Supplementary Figure S1). NEP and TENG are typical conserved sites for GH1 family *β-*glucosidases, and these two motifs function in binding to glucosidase substrates. In addition, the glutamate residues E^168^ and E^354^ within these motifs are crucial for catalytic function, serving as a proton donor and a nucleophile, respectively (Supplementary Figure S1).

### Cloning, expression, and purification of Y50Bg4

**Figure 2:**
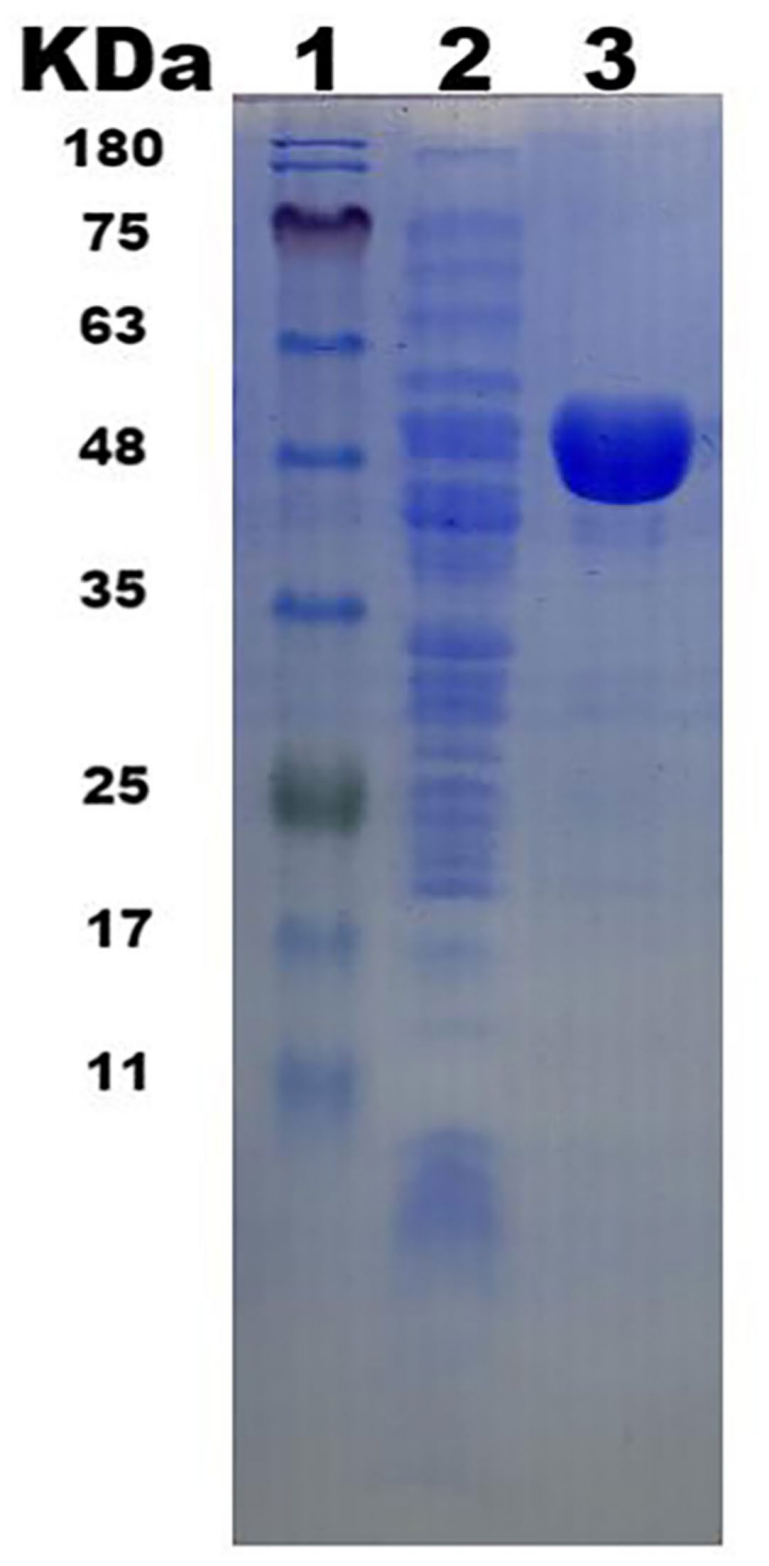
*β*-glucosidase Y50Bg4 SDS-PAGE electropherograms. (1. Protein Marker; 2. Total protein in *E. coli* DH5α/pSHY211-y50bg4 after expression; 3. Purified Y50Bg4 protein).

The gene was successfully expressed in *E. coli* DH5*α* and the recombinant protein with an N-terminus His tag was purified using a Ni-NTA affinity chromatography column. SDS-PAGE analysis revealed a prominent 52 kDa band, consistent with the predicted molecular weight (Figure 2).

### Three-dimensional structure prediction of Y50Bg4

**Figure 3:**
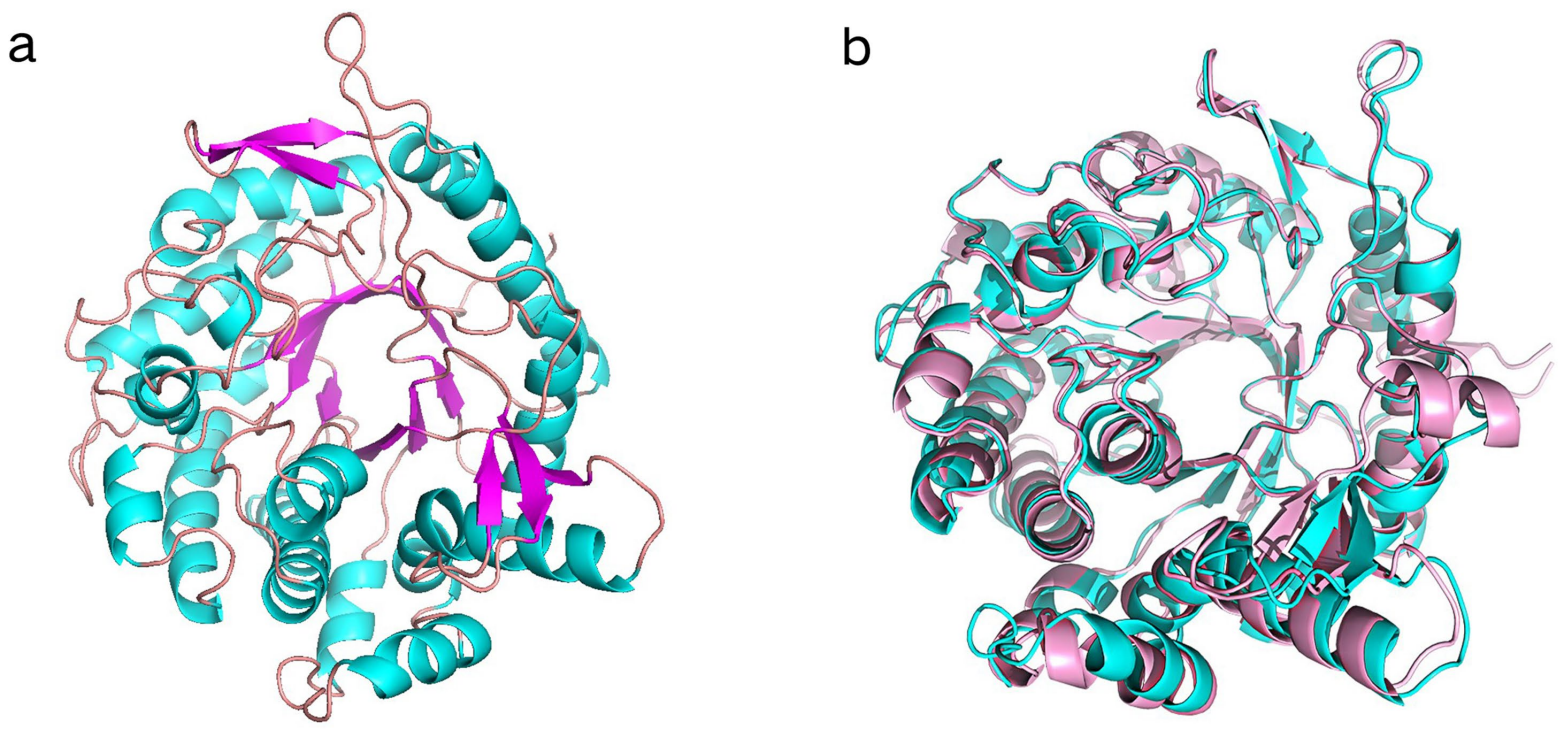
Superimposed results of Y50Bg4 3D structure prediction and B9L147 structure. **(a)** 3D structural model of Y50Bg4. **(b)** Superposition of the Y50Bg4 3D model generated by homology modeling (shown in cyan) onto the B9L147 structure (displayed in pink as a banded model). B9L147 (Uniprot ID) represents a GH1-family *β*-glucosidase originating from *Thermomicrobium roseum*.

The 3D structural model of Y50Bg4 was predicted using AlphaFold, yielding a high-confidence model with a pLDDT score of 97.74 across 440 residues (Figure 3a). The structure displayed a classic 8 α/*β* TIM barrel, characteristic of GH1 enzymes. The predicted structural model of Y50Bg4 was superimposed onto B9L147 through comparative analysis with the three-dimensional structure of B9L147 (UniProt ID: B9L147), resulting in a root-mean-square deviation (RMSD) of 0.477 Å (Figure 3b). B9L147 is a GH1 *β*-glucosidase originating from *Thermomicrobium roseum*, a thermophilic bacterium known for its remarkable tolerance to glucose. Notably, the enzymatic activity of B9L147 was significantly enhanced at glucose concentrations of 1.25–1.5 M, showing an increase of more than 2.5-fold. Even at high glucose concentrations of up to 3 M, B9L147 retained over 100% of its specific activity. Both Y50Bg4 and B9L147 share the characteristic *α/β* TIM barrel domain, a hallmark of GH1 family enzymes.

### Effect of temperature and pH on Y50Bg4 activity and stability

**Figure 4:**
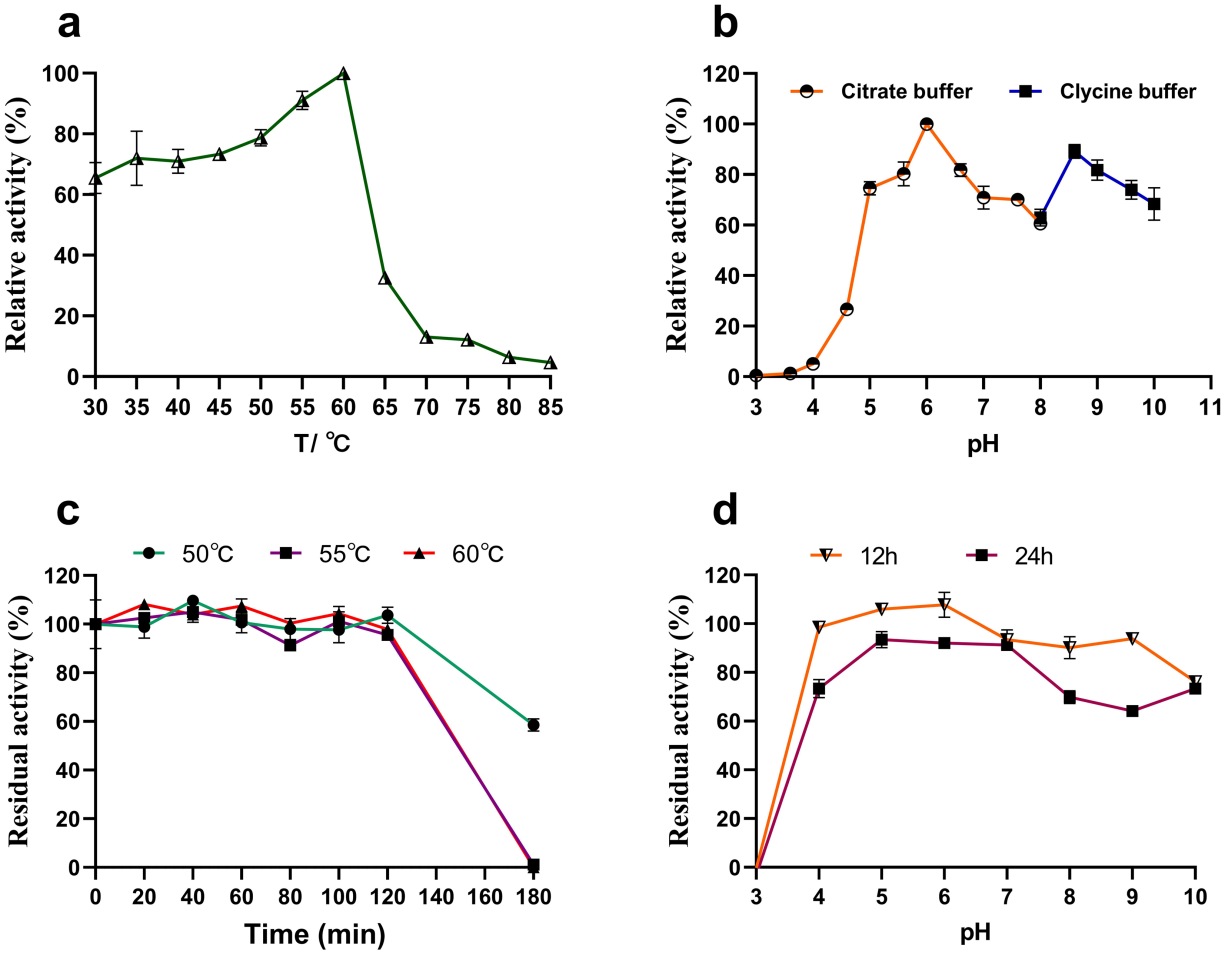
Effect of temperature and pH on the activity and stability of recombinant Y50Bg4. **(a)** Effect of temperature on Y50Bg4 activity. **(b)** Effect of pH on Y50Bg4 activity. **(c)** Effect of incubation at different temperatures (50, 55, and 60°C) for 0, 20, 40, 60, 80, 100, 120, 140, 160, and 180 min on the stability of Y50Bg4. **(d)** Effect of pH on the stability of Y50Bg4. The pH stability test was performed by pre-incubating the enzyme in different buffer systems at 4°C for 12 and 24 h. Values represent the mean of three biological replicates. Error bars represent the mean ± SEM of three biological replicates. The primary activity was taken as 100%.

Y50Bg4 displayed the highest activity at 60°C, maintaining over 60% of its optimal activity between 30 and 60°C (Figure 4a). The optimal pH for activity was determined to be 6.0 and it was assessed at the optimal temperature (Figure 4b). After 2 h of incubation at 50°C, 55°C, and 60°C, the enzyme activity remained at 100%. However, after 3 h of incubation, the activity decreased to about 60% at 50°C and was completely lost at 55°C and 60°C (Figure 4c). Y50Bg4 retained over 60% of its activity across pH 4.0–10.0 after incubation at 4°C for 12 h and 24 h (Figure 4d).

### Effect of glucose concentration on Y50Bg4 activity

**Figure 5:**
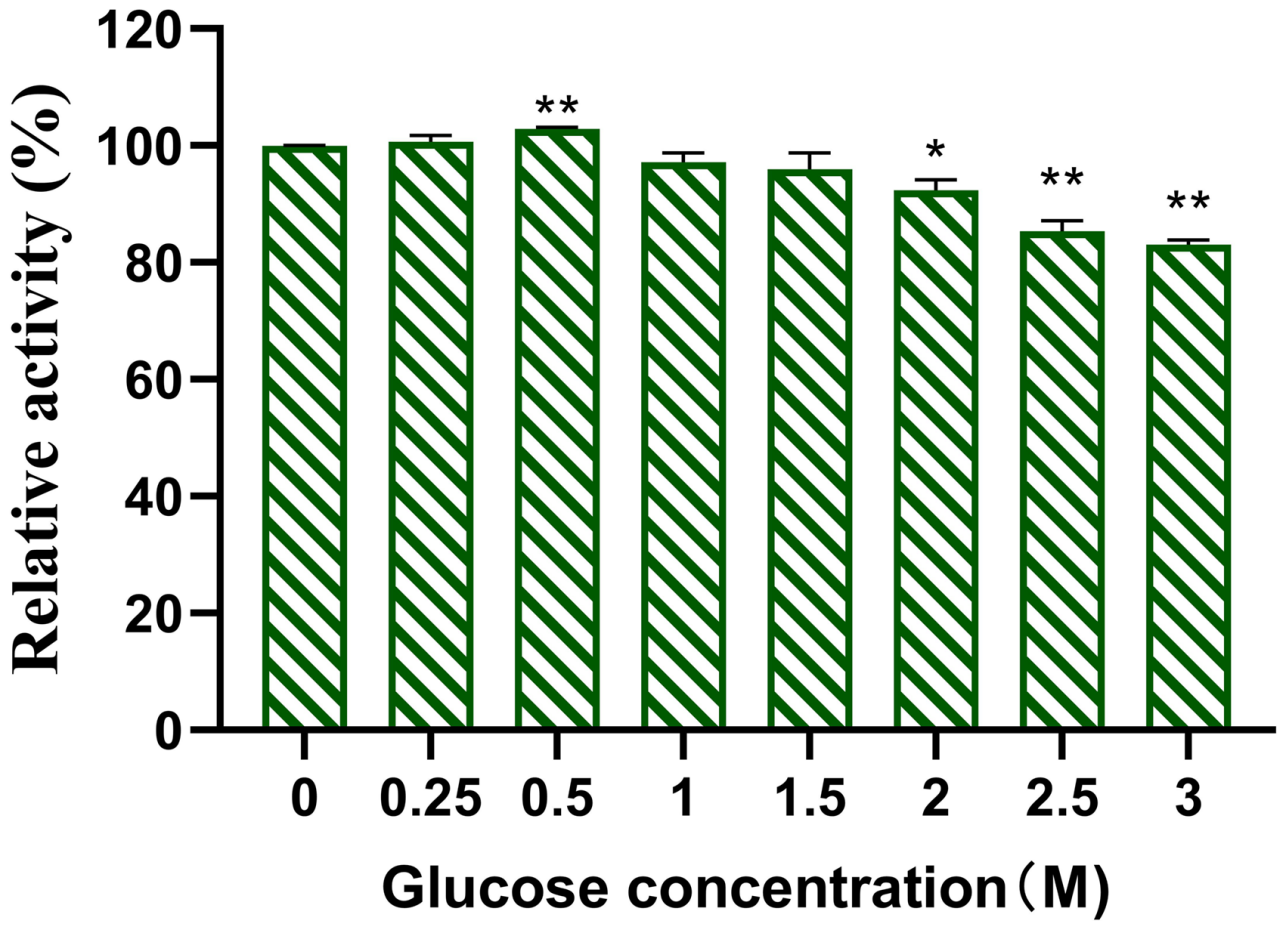
Effect of glucose on recombinant Y50Bg4. The final concentration was determined to be 1 mM using pNPGlc as the substrate. Values represent the mean of three biological replicates. Error bars represent the mean of three biological replicates ± SEM. Original viability was taken as 100%. *p* < 0.01 (**) means the difference is very significant, *p* < 0.05 (*) means the difference is significant.

Competitive inhibition by glucose is a common challenge for *β*-glucosidases and can limit their activity during enzyme-catalyzed hydrolysis. Therefore, high glucose tolerance provides a significant advantage for *β*-glucosidases in certain applications. In this study, the effect of glucose on Y50Bg4 activity was evaluated using 2.5 mM pNPGlc as the substrate. The results showed that Y50Bg4 retained 100% activity at glucose concentrations ranging from 0 to 500 mM and maintained over 80% activity even at 3,000 mM glucose (Figure 5). This remarkable resistance to high glucose concentrations highlights the potential of Y50Bg4 for applications in environments with elevated glucose levels.

### Effect of metal ions and chemical reagents on Y50Bg4 activity

The effects of metal ions and chemical reagents on the activity of Y50Bg4 were assessed at 1 and 10 mM (Table 1). At 1 mM, K^+^, Ca^2+^, Mg^2+^ and Fe^3+^ had minimal effects, while Cu^2+^ (11.79 ± 1.23), Mn^2+^ (7.28 ± 2.37), Pb^2+^ (26.15 ± 1.87) and Ag^+^ (1.94 ± 0.56) caused strong inhibition and Pb^2+^ (26.15 ± 0.56) and Ag^+^ (1.94 ± 0.56) had the highest inhibitory effect. At 10 mM, only K^+^, Ca^2+^, and Mg^2+^ remained non-inhibitory, while the remaining ions reduced activity to nearly 10%. Co^2+^ (0.07 ± 1.15) and Ag^+^ (0.21 ± 0.10) completely suppressed enzyme activity. Chemical reagents such as EDTA (11.86 ± 0.27), Tween-80 (48.83 ± 2.32), PMSF (12.96 ± 1.33), and SDS (9.75 ± 4.59) also significantly reduced activity at 1%.

**Table 1:** Effects of different metal ions and chemical reagents on Y50Bg4 enzyme activity.

	Relative activity/%	
Reagent	1 mM	10 mM
CK	100.00 ± 0.68	
K^+^	102.09 ± 3.02	101.05 ± 2.44
Mg^2+^	97.68 ± 3.06	101.00 ± 0.57
Fe^3+^	99.24 ± 3.60	7.11 ± 1.70**
Ca^2+^	101.14 ± 3.76	104.91 ± 3.99
Zn^2+^	42.99 ± 2.38**	12.54 ± 3.07**
Co^2+^	46.74 ± 6.60**	0.07 ± 1.15**
Cu^2+^	11.79 ± 1.23**	9.79 ± 1.22**
Ag^+^	1.94 ± 0.56**	0.21 ± 0.10**
Mn^2+^	7.28 ± 2.37**	9.48 ± 0.94**
Pb^2+^	26.15 ± 1.87**	11.99 ± 1.50**
Ni^2+^	53.03 ± 4.86**	8.21 ± 1.52**
	0.1%	1%
EDTA	81.32 ± 3.34**	11.86 ± 0.27**
Tween-80	91.23 ± 4.54*	48.83 ± 2.32**
PMSF	25.07 ± 5.49**	12.96 ± 1.33**
SDS	7.53 ± 0.37**	9.75 ± 4.59**
Ethanol	97.39 ± 0.76**	96.62 ± 3.46**

*p* < 0.05 (*) means the difference is significant, *p* < 0.01 (**) means the difference is very significant.

### Substrate specificity and kinetic constants of Y50Bg4

Y50Bg4 hydrolyzed a variety of substrates, including cellobiose, lactose, pNPGlc, and gentiobiose, but was unable to hydrolyze maltose, melibiose, trehalose, raffinose, sucrose, beechwood xylan, corncob xylan, bagasse xylan, CMC-Na, and Avicel (Table 2). The highest activities were observed for cellobiose (31.06 ± 2.25 U/mg) and lactose (23.91 ± 1.74 U/mg), while lower activities were detected for pNPGlc (6.72 ± 0.76 U/mg), pNPGla (4.51 ± 0.02 U/mg), and gentiobiose (1.58 ± 0.16 U/mg).

**Table 2:** Substrate specificities of Y50Bg4.

Substance	Special activity (U/mg)
Cellobiose	31.06 ± 2.25
Lactose	23.91 ± 1.74
*p*-nitrophenyl-*β*-d-glucopyranoside	6.72 ± 0.76
4-nitrophenyl-*β*-d-galactopyranoside	4.51 ± 0.02
*β*-gentiobiose	1.58 ± 0.16
D-(+)-maltose monohydrate	ND
D-(+)-melibiose hydrate	ND
D-(+)-trehalose dihydrate	ND
Raffinose	ND
Beechwood xylan	ND
Sucrose	ND
Corn cob xylan	ND
Bagasse xylan	ND
CMC-Na	ND
Avicel	ND

ND is not detected activity.

The kinetic constants (*K*_m_ and *V*_max_) were determined using Michaelis–Menten non-linear regression (Supplementary Figure S2), and the results have been validated by the Hanes-Woolf method (Supplementary Figure S3). With cellobiose as the substrate, *K*_m_ was 4.69 mg/mL and *V*_max_ was 39.71 μmol/min/mg. For pNPGlc as substrate *K*_m_ was 0.53 mM and *V*_max_ was 20.39 μmol/min/mg, yielding catalytic efficiencies (*K*_cat_/*K*_m_) of 7.44 s^−1^/mg/ml and 33.80 s^−1^/mM, respectively (Table 3).

**Table 3:** Determination of kinetic constants for Y50Bg4.

Substrate	*V*_max_ (μmol/min/mg)	*K*_m_	*K*_cat_ (s^−1^)	*K*_cat_/*K*_m_
Cellobiose	39.71	4.69 mg/mL	34.88	7.44 (s^−1^/mg/mL)
pNPGlc	20.39	0.53 mM	17.91	33.80 (s^−1^/mM)

### Potential use of Y50Bg4 in corn stover hydrolysis

**Figure 6:**
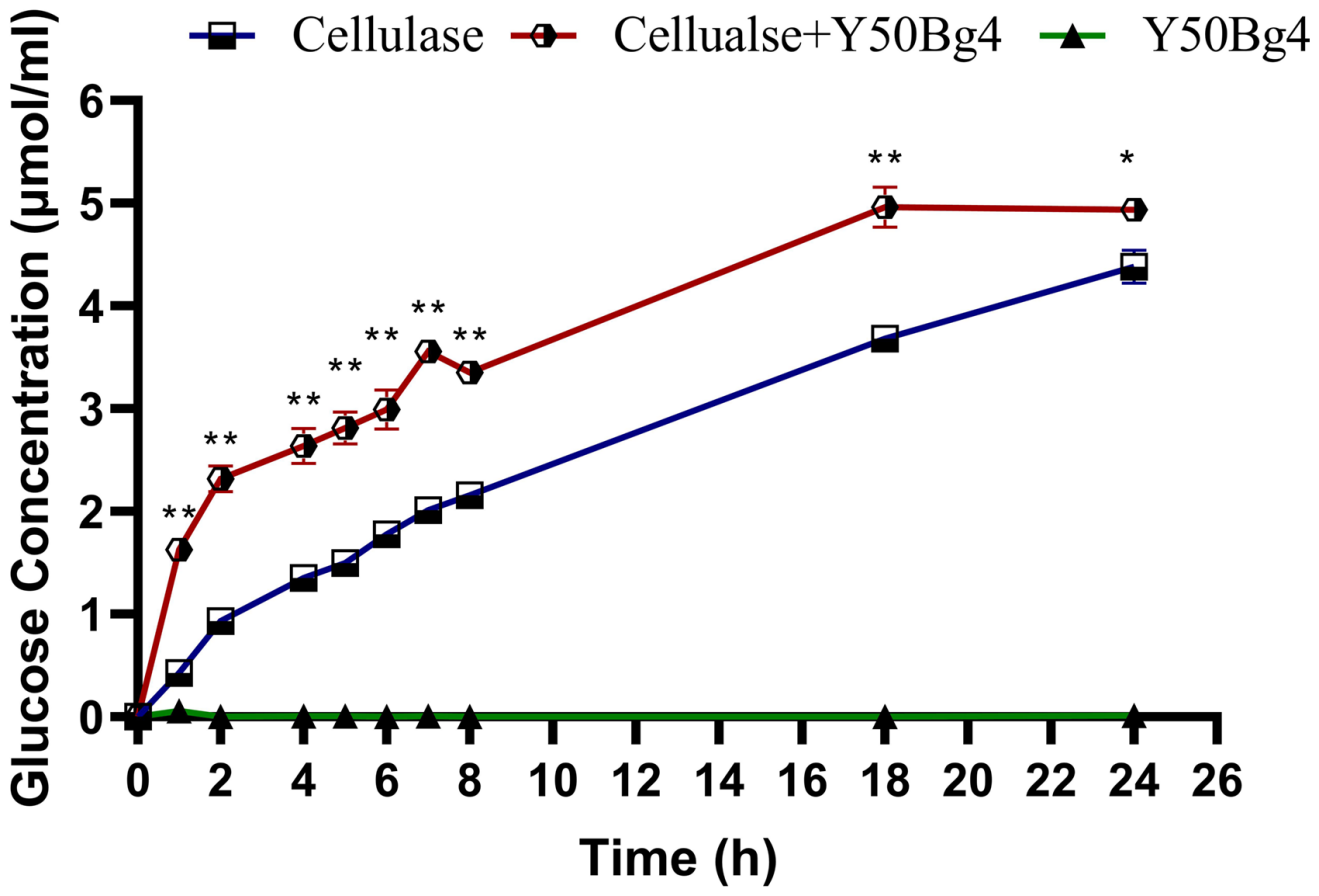
Synergistic degradation of corn stover by Y50Bg4 and commercial cellulases at 60°C. The amount of glucose released following the addition of Y50Bg4 was determined, with the group containing only commercial cellulase serving as the control. Values represent the mean of three biological replicates. Error bars represent the mean ± SEM of three biological replicates. Original viability was taken as 100%. *p* < 0.01 (**) means the difference is very significant, *p* < 0.05 (*) means the difference is significant.

Enzymatic hydrolysis of corn stover was performed to evaluate the potential use of Y50Bg4 in lignocellulose degradation and revealed that Y50Bg4 alone showed no hydrolytic activity. However, the addition of Y50Bg4 to commercial cellulase increased the degradation rate by about 11.6% compared to the hydrolysis rate of the commercial cellulase-only group (Figure 6). This suggests that Y50Bg4 has no hydrolytic activity on corn stover alone but shows good synergistic hydrolysis in cooperation with other cellulases, suggesting its potential as an enzyme-promoted mixture component for converting lignocellulosic biomass into different chemicals.

### Analysis of structural fluctuations and conformational flexibility during simulations

**Figure 7:**
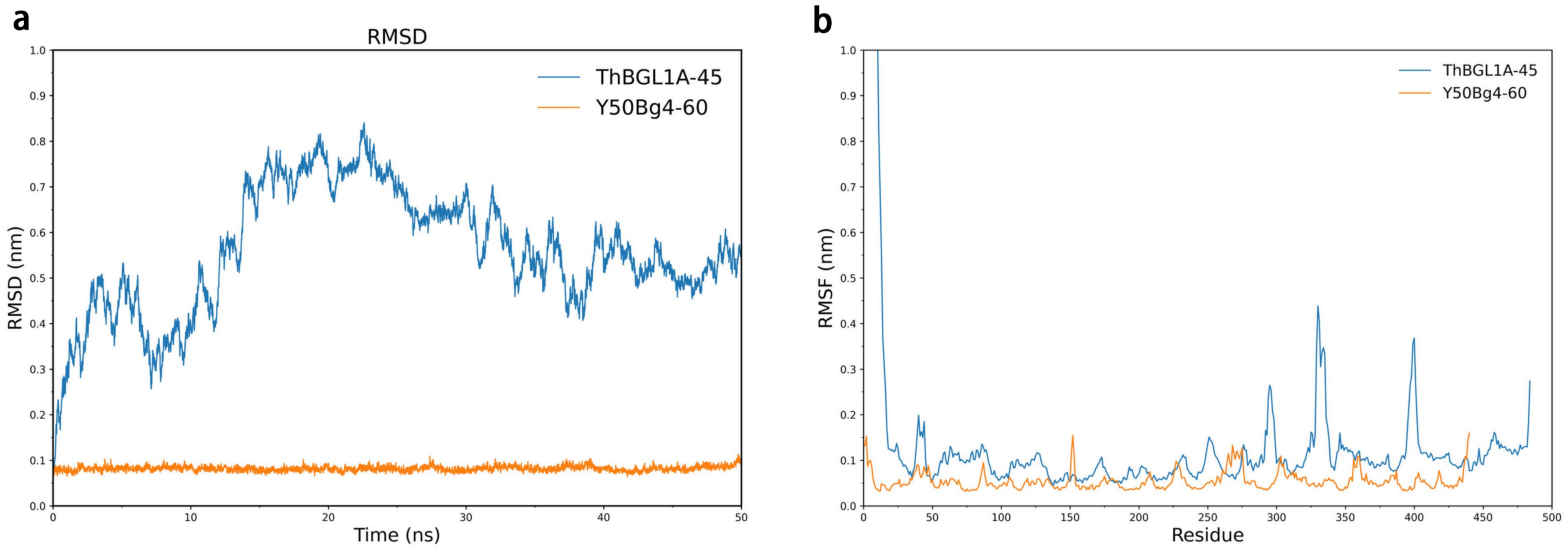
Root means square deviation (RMSD) and RMSF results for Y50Bg4, ThBGL1A. **(a)** RMSD value variation curves as a function of time for backbone atoms in MD simulations for Y50Bg4 (orange line) and ThBGL1A (blue line), respectively. **(b)** Root-mean-square fluctuation (RMSF) values of Cα atoms per residue for Y50Bg4 (orange line) and ThBGL1A (blue line).

The overall stability/equilibrium of Y50Bg4 and ThBGL1A during the molecular dynamics simulation process were evaluated by calculating the backbone RMSD as a function of time for both simulated systems. As shown in Figure 7a, the skeleton RMSD of the Y50Bg4 system remained relatively stable, fluctuating minimally throughout the simulation. In contrast, the ThBGL1A system exhibited greater fluctuations, requiring approximately 40 ns to stabilize before maintaining relatively steady values. Consequently, the MD trajectories between 10–50 ns for both systems were used as equilibrium trajectories for subsequent analysis. During the simulation, ThBGL1A consistently displayed higher RMSD values than Y50Bg4, indicating greater structural fluctuations and conformational changes compared to its initial structure. This increased flexibility of ThBGL1A might explain why Y50Bg4 demonstrates superior thermal stability. To investigate the conformational dynamics root-mean-square fluctuations (RMSF) of C*α* atoms were calculated. As illustrated in Figure 7b, both proteins showed similar RMSF patterns in some regions, suggesting partial structural and interface similarity. However, Y50Bg4 exhibited lower RMSF values in most regions compared to ThBGL1A, aligning with the observed RMSD trends. Notably, two loop regions in ThBGL1A (residues 325–350 and 390–410) displayed significantly higher RMSF values than corresponding regions in Y50Bg4. These findings suggest that ThBGL1A possesses greater global conformational flexibility than Y50Bg4.

## Discussion

Phylogenetic analysis revealed that the Y50Bg4 enzyme clusters closely with *β*-glucosidases from *Caloramator quimbayensis*. The study identified *Caloramator quimbayensis* as an anaerobic, moderately thermophilic bacterium isolated from terrestrial hot springs (Rubiano-Labrador et al., 2013). This bacterium grows within a temperature range of 37–55°C and pH 6.0–8.0, with optimal growth conditions at 50°C and pH 7.0, suggesting that Y50Bg4 may also exhibit thermophilic properties. It does not necessarily indicate that *Caloramator quimbayensis* is the direct origin of the Y50Bg4 enzyme. The actual source of Y50Bg4 remains unclear and may require further investigation, such as screening genomic or metagenomic data from environmental samples to identify its natural host. Y50Bg4 belongs to the GH1 family of *β*-glucosidases, which comprises most known *β*-glucosidases. GH1 enzymes are generally more glucose-tolerant than those from the GH3 family, playing a crucial role in cellulose saccharification (Shipkowski and Brenchley, 2005; Ketudat Cairns and Esen, 2010; de Almeida et al., 2020).

The conservation of the *α/β* TIM barrel fold within the GH1 family highlights its functional importance. This structural motif serves as a scaffold for the precise positioning of key catalytic residues, ensuring efficient substrate binding and hydrolysis (Zechel and Withers, 2000; Nagano et al., 2002). Specifically, the conserved glutamate residues E^168^ and E^354^, located within the active site, are known to function as proton donors and nucleophiles, respectively. The low RMSD value of 0.477 Å further emphasizes the close structural relationship between Y50Bg4 and B9L147. Such a small deviation suggests that the overall folding and architecture of the two enzymes are highly conserved, reflecting their shared functional mechanisms. The minor differences observed in the loop regions may contribute to variations in substrate specificity or thermostability, warranting further investigation.

Garbin et al. (2021) reported that *β*-glucosidase from the thermophilic fungus *Thermoascus crustaceous* remained stable at 30–55°C after 1 h of incubation, with about 60% residual activity at 60°C. In comparison, Toyama et al. (2018) found that the *β*-glucosidase AmBgl-LP from the Amazonian Lake Pollack metagenome maintained 60% residual activity after 30 min at 40°C but lost all activity after 10 min at 50°C. In contrast, as shown in Table 4, Y50Bg4 demonstrated better thermal stability, a broader pH tolerance, and higher efficiency in degrading naturally crystalline, structurally complex cellulose.

**Table 4:** Enzymatic properties as well as kinetic parameters of Y50Bg4 compared to other *β*-glucosidases.

Enzyme name	Organism	*K*_m_ (mM)	*V*_max_ (μmol/min/mg)	*k*_cat_ (s^−1^)	Optimal temperature (°C)	pH Range	Glucose Tolerance (M)	Reference
Y50Bg4	Hot spring metagenome	0.53	20.39	17.91	70°C	5.0–10.0	80% relative activity at 3.0 M	This study
AmBgl-LP	Freshwater metagenome	5.38	ND	33.7	45°C	5.5–6.5	ND	Toyama et al. (2018)
ND	Black plum seeds	0.58	90.2	ND	55°C	4.0–6.0	50% relative activity at 0.08 M	Bi et al. (2019)
PABGL	*Pseudoalteromonas* sp.	2.023 ± 0.1553	7.357 ± 0.1627	ND	40°C	5.0–10.0	20% relative activity at 0.02 M	Qu et al. (2020)
ND	*Thermoascus crustaceus*	ND	ND	ND	65°C	4.0–8.8	30% relative activity at 0.1 M	Garbin et al. (2021)
Bgl1973	*Leifsonia* sp. ZF2019	0.22	44.44	57.78	50°C	6.6–9.0	30% relative activity at 2.0 M	He et al. (2022)
Bgl3	*Neofusicoccum parvum*	1.18	28.8	ND	60°C	3.0–4.0	10% relative activity at 0.8 M	Singh et al. (2023)
CelGH3_f17	Soda lake metagenomic	ND	ND	ND	50°C	7.5–10.5	40% relative activity at 0.5 M	Jeilu et al. (2024)

Kinetic parameters (*K*_m_, *V*_max_, *k*_cat_) were measured using pNPGlc as a substrate under standard assay conditions. ND indicates no mention.

Most microbial *β*-glucosidases are competitively inhibited by glucose, with inhibition constant (*K*_i_) values typically between 100 and 500 mM (Mamma et al., 2004; Karnchanatat et al., 2007). However, some *β*-glucosidases, such as Unbgl1A from soil metagenomic source, tolerate glucose concentrations up to 1,500 mM, with activation observed at 300 mM (Lu et al., 2013). Y50Bg4 exhibits higher glucose tolerance than most of the *β*-glucosidases, making it an ideal candidate for industrial processes requiring robust enzyme activity under high glucose concentrations. The glucose tolerance of Y50Bg4 may be linked to its active site architecture and substrate binding mode. Beta-glucosidases from the GH1 family have been reported to be generally glucose-tolerant, with a narrower and deeper active site at the *α/β* 8 barrel, whereas the majority of glucose-intolerant beta-glucosidases have been studied under the GH3 family, which has a shallower lumen (de Giuseppe et al., 2014). The binding of glucose to the shallow lumen easily leads to competitive inhibition with the substrate, while a wider or more flexible substrate-binding pocket can reduce the steric hindrance caused by glucose binding (Sengupta et al., 2023). Y50Bg4 showed sensitivity to metal ions and chemical reagents, of which Ag^+^, Zn^2+^, and SDS act as strong inhibitors, similar to other *β*-glucosidases (Zollner, 1989). However, Ca^2+^ and Mg^2+^ stabilized its activity, aligning with findings for other GH1*β*-glucosidases. The inhibitory effects of EDTA suggest that Y50Bg4 depends on metal ions for activity. These results are consistent with studies on *β*-glucosidases such as BGLA from *Alteromonas* sp. L82 and its F171W mutant (Sun et al., 2020), where SDS disrupted the enzyme three-dimensional structure and catalytic domain integrity (Geiger et al., 1999).

To further determine the hydrolytic activity of Y50Bg4 on lactose, the results of the optimal temperature (60°C) and optimal pH (6.6) of Y50Bg4 determined using lactose as the substrate were almost the same as those determined when cellobiose was used as the substrate (Supplementary Figure S4). Y50Bg4 efficiently hydrolyzed *β*-(1 → 4) glycosidic bonds but failed to cleave *α*-glycosidic linkages in sucrose or maltose and complex polysaccharides such as xylan, CMC-Na, and Avicel, whose long chains of polysaccharides hinder the activity of BGLs due to the complexity of the substrates. In summary, Y50Bg4 is a multifunctional enzyme with the activity of both *β*-glucosidase and *β*-galactosidase pairs. This result is consistent with Xie et al. (2020) who reported that the *β*-glucosidase Igag_0940 derived from hyperthermophile *Ignisphaera aggregans* also showed activity toward cellobiose, lactose, and is also a multifunctional glycoside hydrolase of GH1. Furthermore, Deng et al. (2020) reported that from the deep-sea bacterium *Bacillus* sp. D1 also screened and cloned a novel *β*-glucosidase, BglD1, with high *β*-galactosidase and *trans* glycosidic activities. This is also a feature of the GH1 family of glucosidases, which mostly have both *β*-galactosidase activity and *β*-glucosidase activity (Cantarel et al., 2009). Additionally, Y50Bg4 exhibited high catalytic efficiency for the synthesis of the substrate pNPGlc, which is a key factor in the development of all *β*-glucosidases (Rani et al., 2015; Akram et al., 2016). However, the catalytic efficiency of cellobiose is crucial in determining the efficient degradation of biomass.

**Figure 8:**
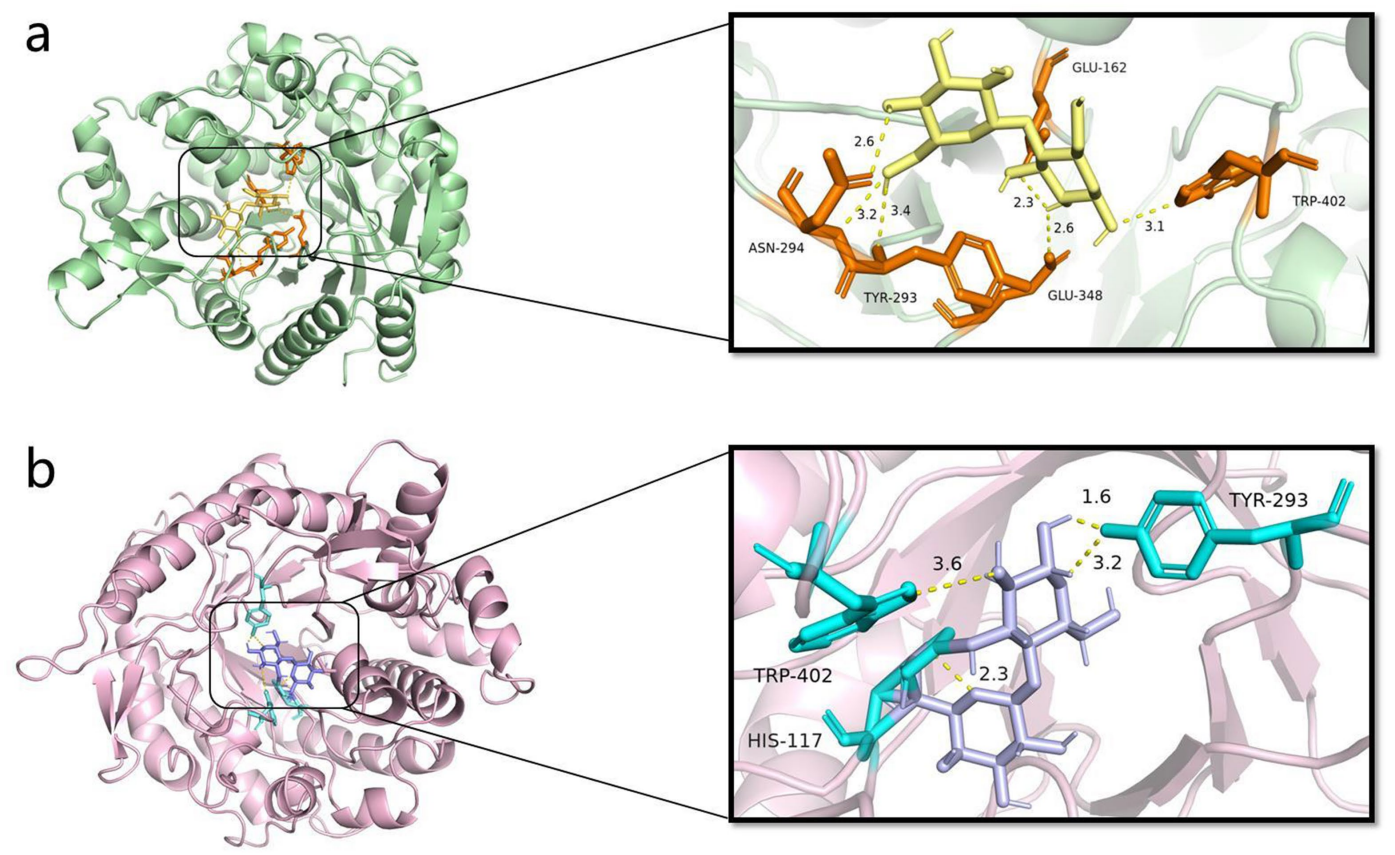
Y50Bg4 docking results with ligand. **(a)** Y50Bg4 docking results with cellobiose, yellow represents cellobiose, orange represents residues interacting with the ligand, and yellow dashed line represents hydrogen bonding. **(b)** Y50Bg4 docking results with lactose, purple represents lactose, cyan represents residues interacting with the ligand, and yellow dashed lines represent hydrogen bonds.

The molecular docking of Y50Bg4 with cellobiose and *α*-lactose revealed successful binding of both substrates. Ten docked conformations with negative binding free energies were obtained for each ligand, indicating favorable interactions. The lowest binding free energy conformation for cellobiose was −7.5 kcal/mol (Figure 8a), while for *α*-lactose, it was −7.4 kcal/mol (Figure 8b), suggesting strong binding affinity for both substrates. Intermolecular interactions, especially hydrogen bonding, played an important role in the formation of enzyme-substrate complexes. In the Y50Bg4-cellobiose complex, six hydrogen bonds were observed, involving residues Asn-294, Tyr-293, Glu-348, Trp-402, and Glu-162. In contrast, the Y50Bg4-lactose complex formed only four hydrogen bonds, involving residues Trp-402, Tyr-293, and His-117. The higher number of hydrogen bonds with cellobiose suggests that Y50Bg4 interacts more efficiently with this substrate, indicating a substrate preference likely due to better structural compatibility. Notably, residues Trp-402 and Tyr-293 were involved in binding both substrates, consistent with findings by Khairudin and Mazlan (2013), who reported Trp-402 as a conserved residue involved in the interaction of proteins with fibrous disaccharides and polysaccharides. This supports the notion that these residues are critical for substrate recognition and catalysis, highlighting Y50Bg4 potential in biodegradation and glycolysis. Y50Bg4 may hydrolyze both α-lactose and cellobiose, which is consistent with our experimental results, suggesting Y50Bg4 is more efficient at hydrolyzing cellobiose. This makes it a promising candidate for enzymatic hydrolysis of lignocellulose into fermentable sugars, a key step in biofuel production. Multifunctional glycoside hydrolases like Y50Bg4 are valuable for the saccharification of cellulose and hemicellulose (Gruninger et al., 2014).

Kinetic simulations revealed that Y50Bg4 exhibited lower structural fluctuations and greater conformational stability compared to the mesophilic enzyme ThBGL1A. It is well established that protein thermal stability is often associated with reduced conformational flexibility. The thermal stability of proteins is usually associated with their reduced flexibility (or increased rigidity), and rigidity protects proteins from unfolding, degradation by proteolytic hydrolysis, and chemical denaturation at high temperatures, implying that inhibition of fluctuations within the protein and the mobility may be responsible for its ability to resist thermal denaturation (Kamerzell and Middaugh, 2008; Karshikoff et al., 2015; Quezada et al., 2017). In the present study, we observed that the overall fluctuation of Y50Bg4 was lower than that of its homologous counterpart ThBGL1A. Supporting the theory that reduced flexibility enhances thermostability. Therefore, the greater thermal stability of Y50Bg4 can be attributed to its higher rigidity and less conformational flexibility than mesothermal enzyme. In addition to the above, the thermostability of Y50Bg4 is also likely attributed to several structural features commonly observed in thermostable enzymes. First, sequence analysis reveals a high proportion of hydrophobic residues, which may contribute to the formation of a stable hydrophobic core. This core helps maintain the enzyme’s tertiary structure under high temperatures. Second, the presence of salt bridges and hydrogen bonds in key regions of the protein could enhance its resistance to thermal denaturation. These interactions are known to stabilize proteins by reducing conformational flexibility at elevated temperatures. In summary, with its unique stability, high substrate specificity, and thermal adaptability, Y50Bg4 holds significant promise for industrial biocatalysis applications. Its efficient catalytic mechanism and resilience under harsh conditions make it an ideal candidate for processes such as biofuel production and biomass conversion.

## Conclusion

The *β*-glucosidase gene *y50bg4* from the Tengchong Thermal Sea metagenomic source was successfully cloned and heterologously expressed in *E. coli*. Detailed characterization of the recombinant *β*-glucosidase Y50Bg4 revealed notable enzymatic properties and provided insights into its thermostable mechanism. The results showed that Y50Bg4 has excellent thermal stability, making it a promising candidate for applications in high-temperature industrial processes. Structural analysis showed that Y50Bg4 has greater rigidity and more compact conformation compared to ambient enzymes4. Notably, Y50Bg4 is a bifunctional enzyme with both *β*-glucosidase and *β*-galactosidase activities, confirmed through molecular docking studies. In addition, its exceptional glucose tolerance surpasses that of most GH1-family *β*-glucosidases, making it an ideal candidate enzyme for use in cellulose degradation saccharification processes as well as biofuel production.

## Data Availability

The datasets presented in this study can be found in online repositories. The names of the repository/repositories and accession number(s) can be found: https://www.ncbi.nlm.nih.gov/genbank/, PQ827568.

## Footnotes

^1^ https://img.jgi.doe.gov/cgi-bin/mer/main.cgi

^2^ http://blast.ncbi.nlm.nih.gov/Blast.cgi

^3^ https://services.healthtech.dtu.dk/services/SignalP-5.0/

^4^ http://web.expasy.org/protparam/

^5^ https://www.ncbi.nlm.nih.gov/protein

^6^ https://alphafold.com/

^7^ http://espript.ibcp.fr/ESPript/ESPript/index.php

^8^ https://pubchem.ncbi.nlm.nih.gov
