# Supplementary material for: Characterization, thermostable mechanism, and molecular docking of a novel glucose-tolerant β-glucosidase/β-galactosidase from the GH1 family isolated from Rehai hot spring
Source: Front Microbiol. 2025 Apr 11;16:1559242. doi: 10.3389/fmicb.2025.1559242 (PMC12023779; doi:10.3389/fmicb.2025.1559242)
Supplement: Supplementary file 1 [file Data_Sheet_1.docx]

**Supplementary materials**

**List of the supporting materials:**

**4 figure: Supplementary Figure S1，Supplementary Figure S2, Supplementary Figure S3, Supplementary Figure S4**


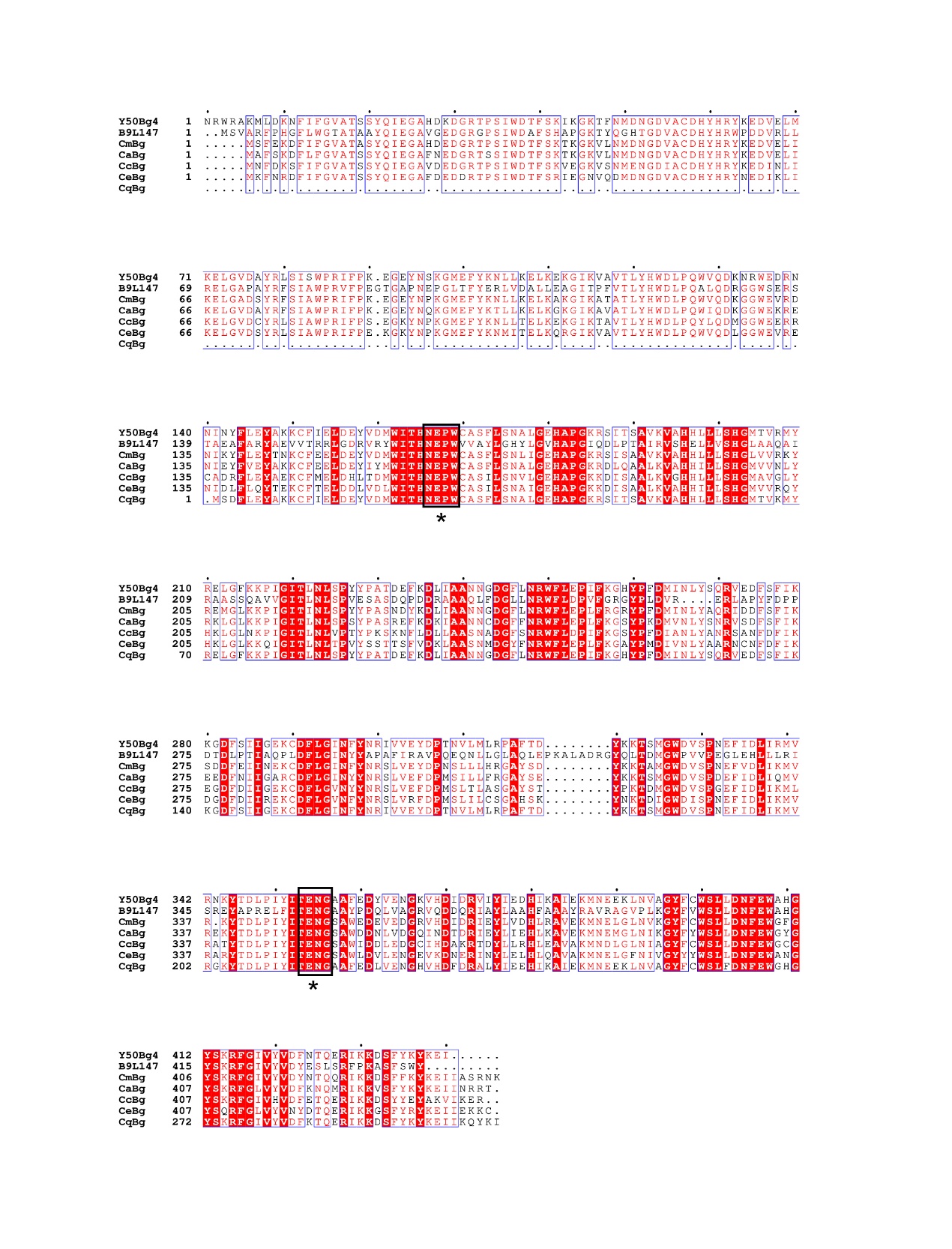
**Supplementary Figure S1** **Sequence alignment of β-glucosidases.**

Similar sequences are marked with boxes, identical sequences are highlighted in red. the 'NEP' and 'TENG' conserved regions are marked with black boxes the bottom pattern is marked with *. The sequences used in this alignment are listed below with their respective protein IDs and source organisms: B9L147 (*Thermomicrobium roseum* ID: WP_015922700.1), CmBg (*Caloramator mitchellensis* ID: WP_200956812.1), CaBg (*Caloramator australicus* ID: WP_008907518.1), CcBg (*Clostridium* sp. CS001 ID: WP_226261516.1), CeBg：*Clostridium* sp. E14 ID: WP_264851258.1), CqBg (C*aloramator quimbayensis* ID: WP_278305990.1).

**
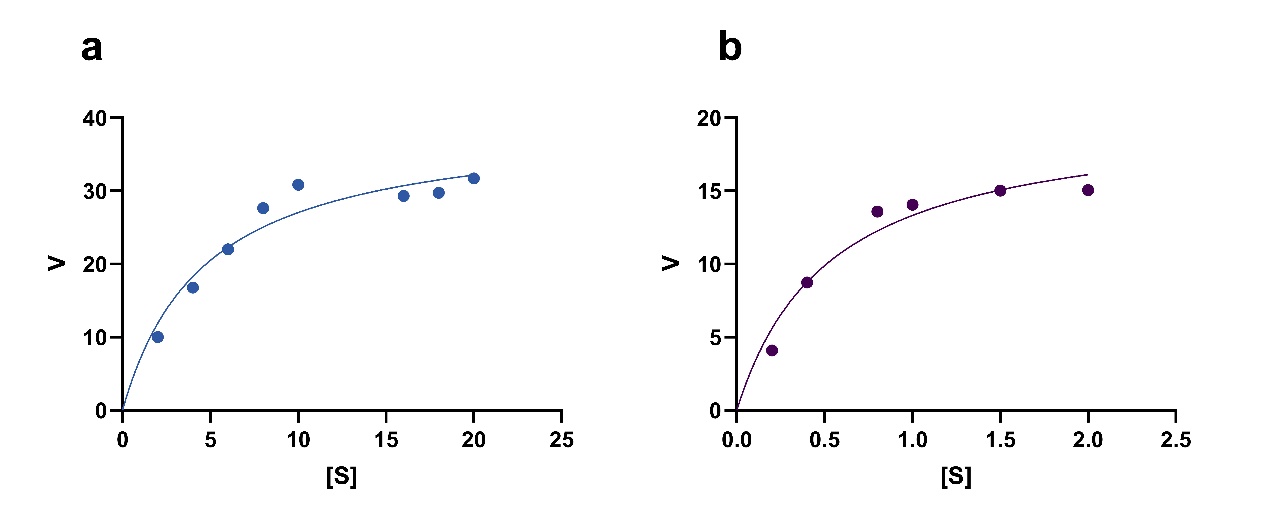
**

**Supplementary Figure S2** **Michaelis-Menten plot of Y50Bg4 β-glucosidase**

**The reaction rate (*v*) of Y50Bg4 was measured at various substrate concentrations ([*S*])**

**(a) Cellobiose as the substrate. *V*_max_ of 39.71μmol/min and *K*_m_ of 4.69 mg/mL.**

**(b) pNPGlc as substrate. *V*_max_ of 20.39μmol/min and *K*_m_ of 0.53 mM.**


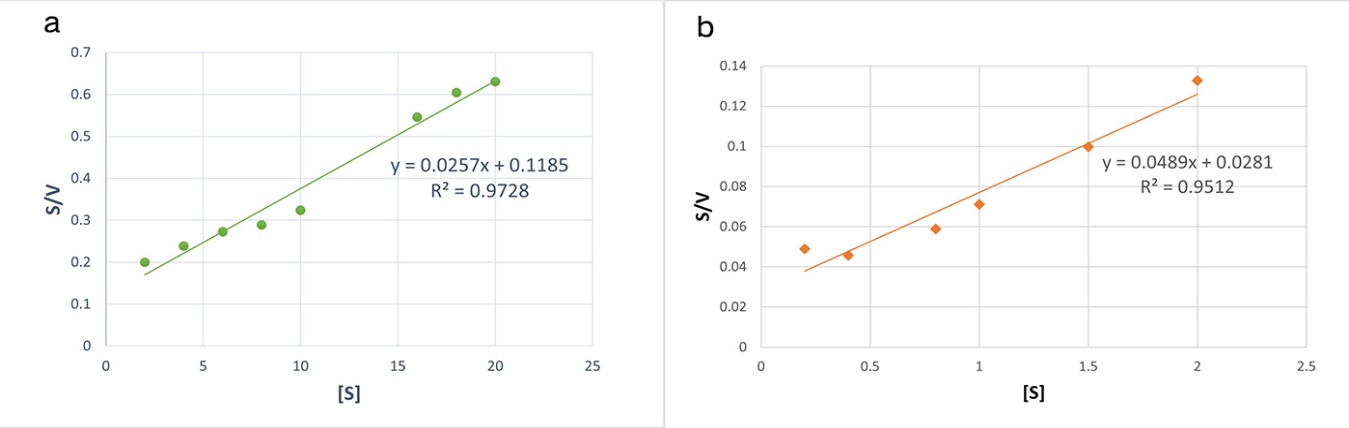


**Supplementary Figure S3** **Hanes-Woolf plot of Y50Bg4 β-glucosidase.**

**The Hanes-Woolf plot depicts [S]/v versus [S] , where [S] is the substrate concentration and v is the reaction velocity.(a) Cellobiose as substrate (y=0.0257x+0.1185 R^2^=0.9728).**

***V*_max_ of 38.91μmol/min and *K*_m_ of 4.62 mg/mL.**

**(b) pNPGlc as substrate (y=0.0489x+0.0281 R^2^=0.9512).**

***V*_max_ of 20.45μmol/min and *K*_m_ of 0.57 mg/mL.**


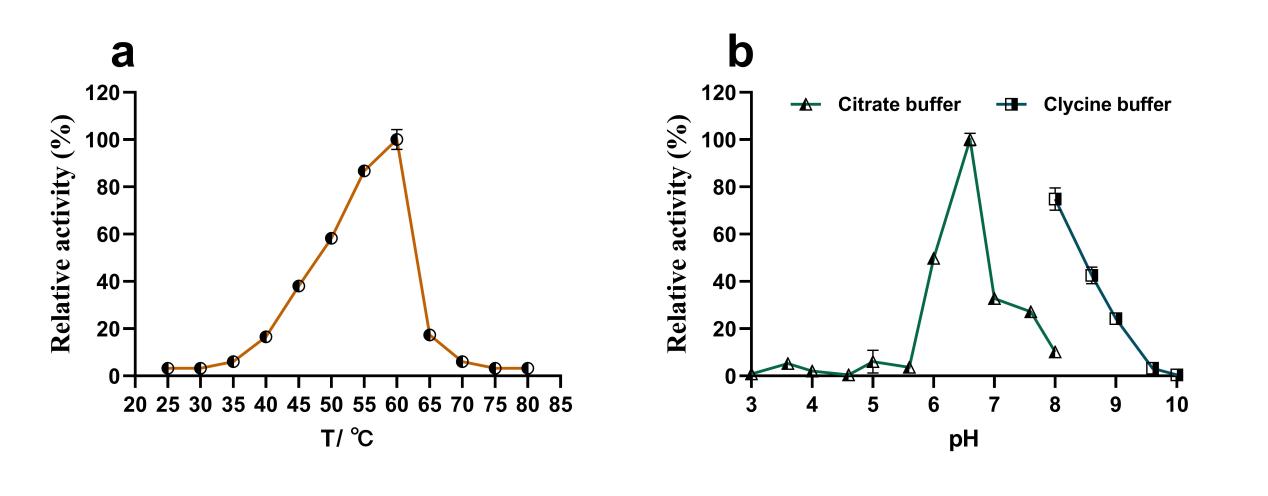


**Supplementary Figure S4 Effect of temperature and pH on the activity and stability of recombinant Y50Bg4 with lactose as substrate.**

**(a) Y50Bg4 optimum temperature; (b) Y50Bg4 optimum pH.**

**Values represent the mean of three biological replicates. error bars represent the mean ± SEM of three biological replicates. the primary activity was taken as 100%.**
